# Common Carotid Artery Diaphragm presenting as transient syncope

**DOI:** 10.1016/j.radcr.2021.04.076

**Published:** 2021-06-08

**Authors:** Fatima zahrae LAAMRANI, Laila Jroundi, Hiba Zahi, Mohamed Lahkim

**Affiliations:** Department of Emergency Radiology, Ibn Sina Hospital, 19 Rue Camelia, Secteur 9, Rabat**,** Morocco

**Keywords:** Common Carotid Artery Diaphragm, Doppler, CT angiography, ICA, internal carotid artery, CCA, Common carotid artery, CDU, Carotid duplex ultrasound, CTA, Computed tomography angiography, FMD, Fibro muscular dysplasia

## Abstract

Carotid diaphragm usually can cause artery stenosis essentially affecting the internal carotid artery (ICA) beyond the bulb segment.  Patients are often middle aged and the onset of symptoms typically begins with syncopes leading to ischemic stroke.  More than 50 cases have been reported in which all lesions showed as stenosis located in the ICA, in this article, we report a rare common carotid artery (CCA) diaphragm revealed in a 59 year-old female suffering from transient syncopes. This is the third case reported in the literature.

## Case report

A 48-year-old Moroccan, nonsmoking female, was hospitalized for recurring transient syncope. She had a history of hypertension and balanced diabetes, but no history of heart disease, trauma or dyslipidemia. Physical examination was normal apart from a bruit at right carotid auscultation.

Carotid duplex ultrasound (CDU) showed a right thin carotid diaphragm located in the proximal CCA (common carotid artery), (Fig. A). High peak systolic velocities of 250 cm/second with turbulent color jet were detected at the level of proximal carotid web (Fig. B), suggesting a greater stenosis then 70%. Velocities in the left internal carotid artery were normal, excluding stenosis. An aneurismal dilatation with approximately 5 cm length was also detected at the lesion. Subsequently under further evaluation, it was confirmed by computed tomography angiography (CTA) (Fig. C and D) showing a transverse, low density strip on proximal part on the right CCA causing lumen stenosis, with post aneurismal enlargement.

**Fig. A:**
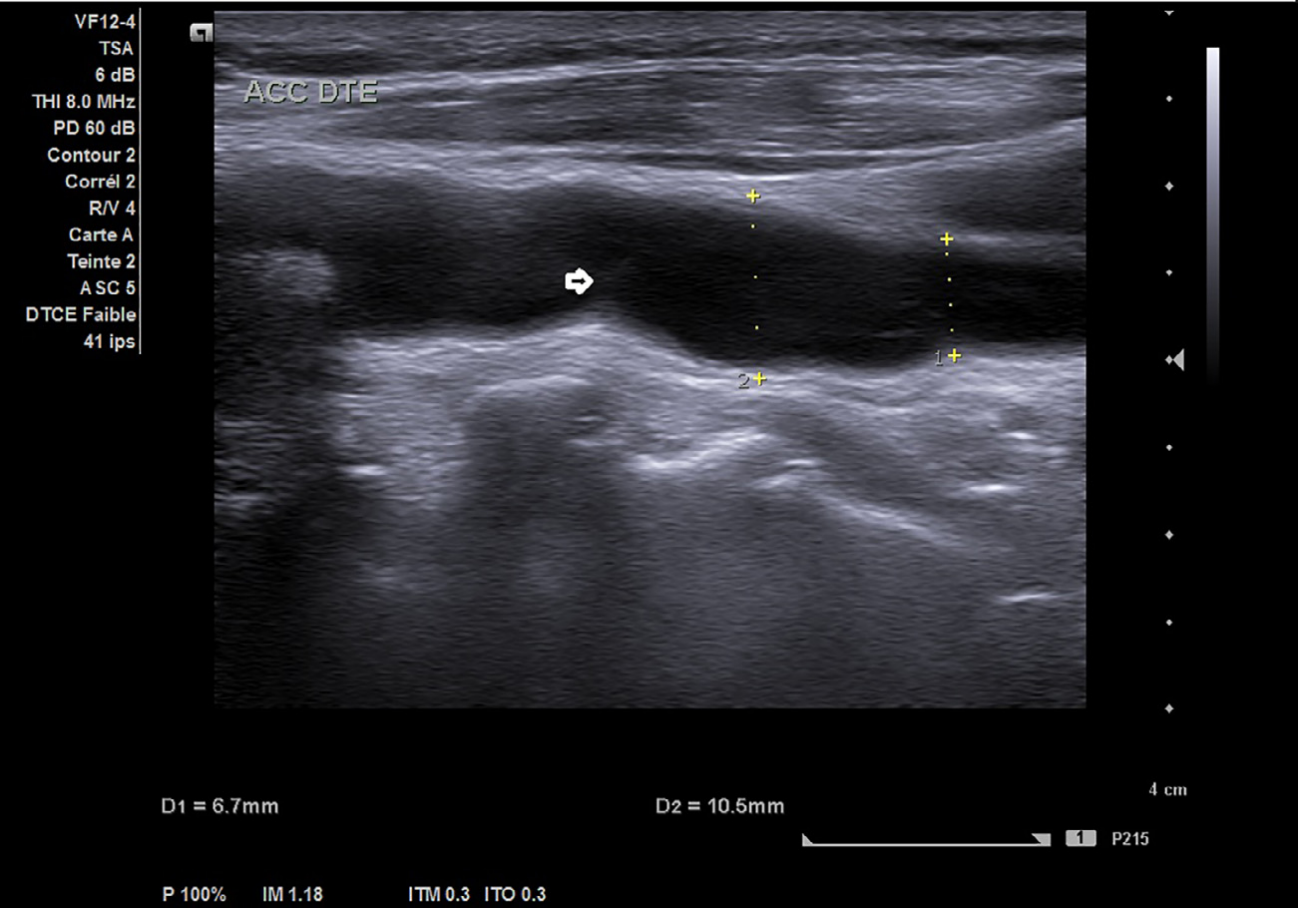
Color Doppler ultrasound scan showed an obviously localized dilation (arrow) with a thin diaphragm with a small central lumen on the distal part of CCA.

**Fig. B:**
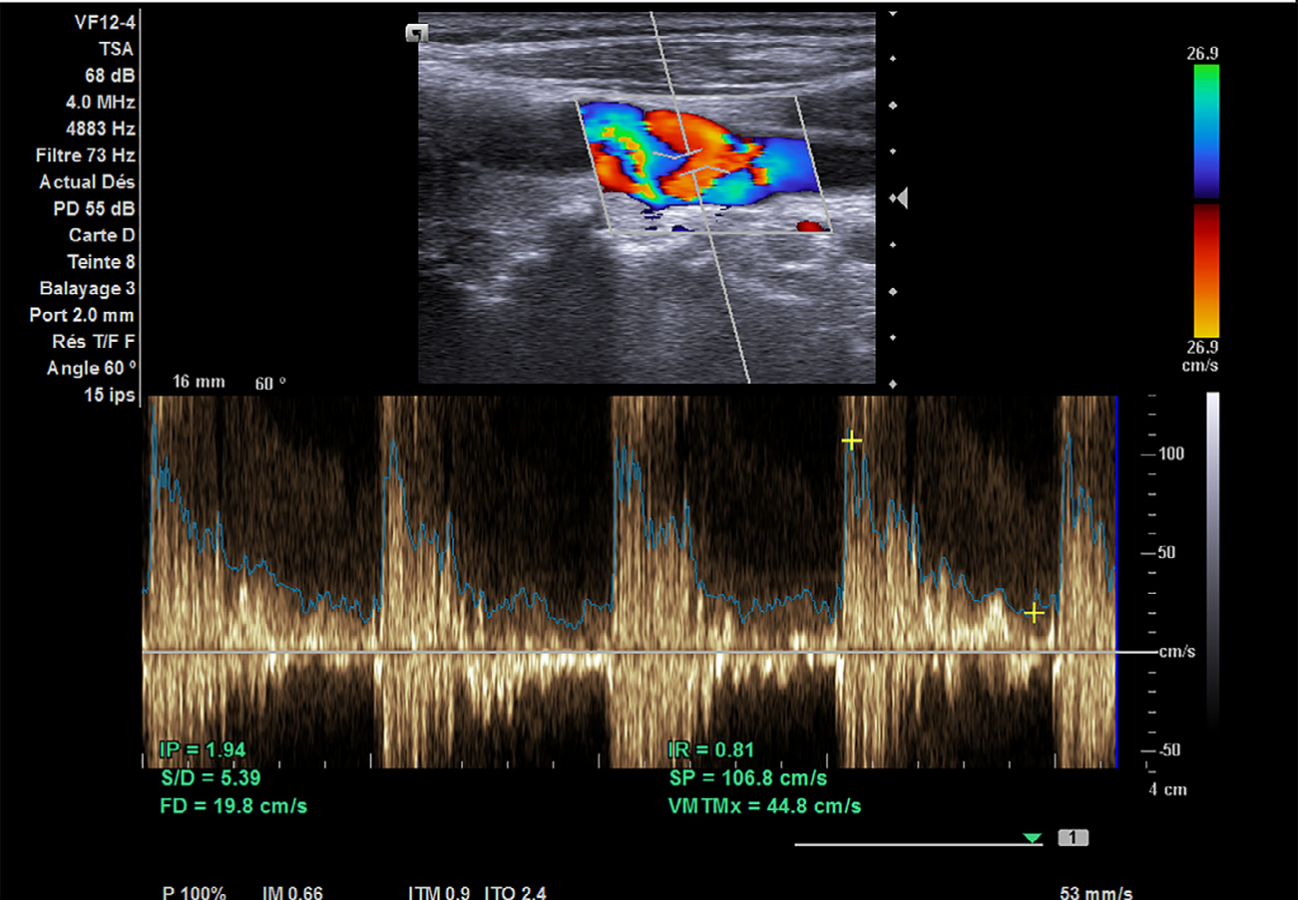
CDU showed turbulent color jet through this lumen of high velocity blood flow of 250cm/s (arrow). P^).

**Fig. C:**
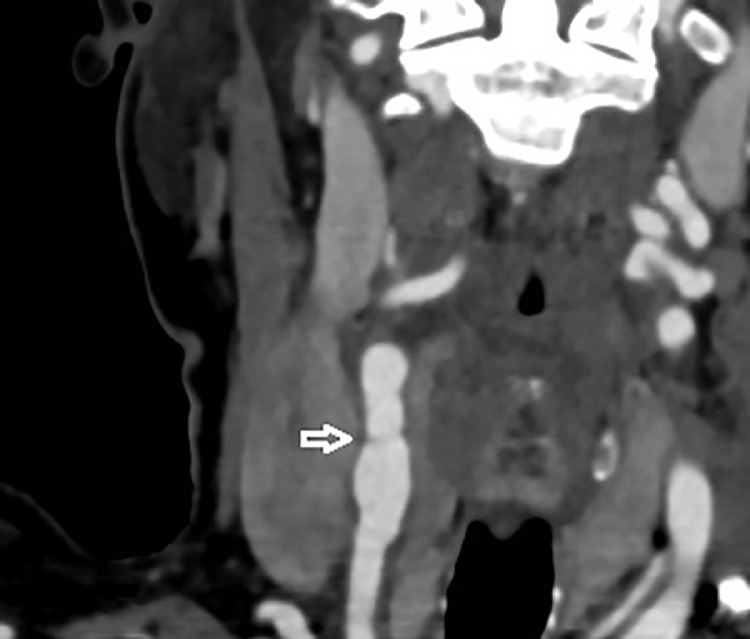
CTA showed a transverse low desnity strip ( arrow), causing luman stenosis with proximal post CCA aneurysmal enlargement on lower part of right.

**Fig. D:**
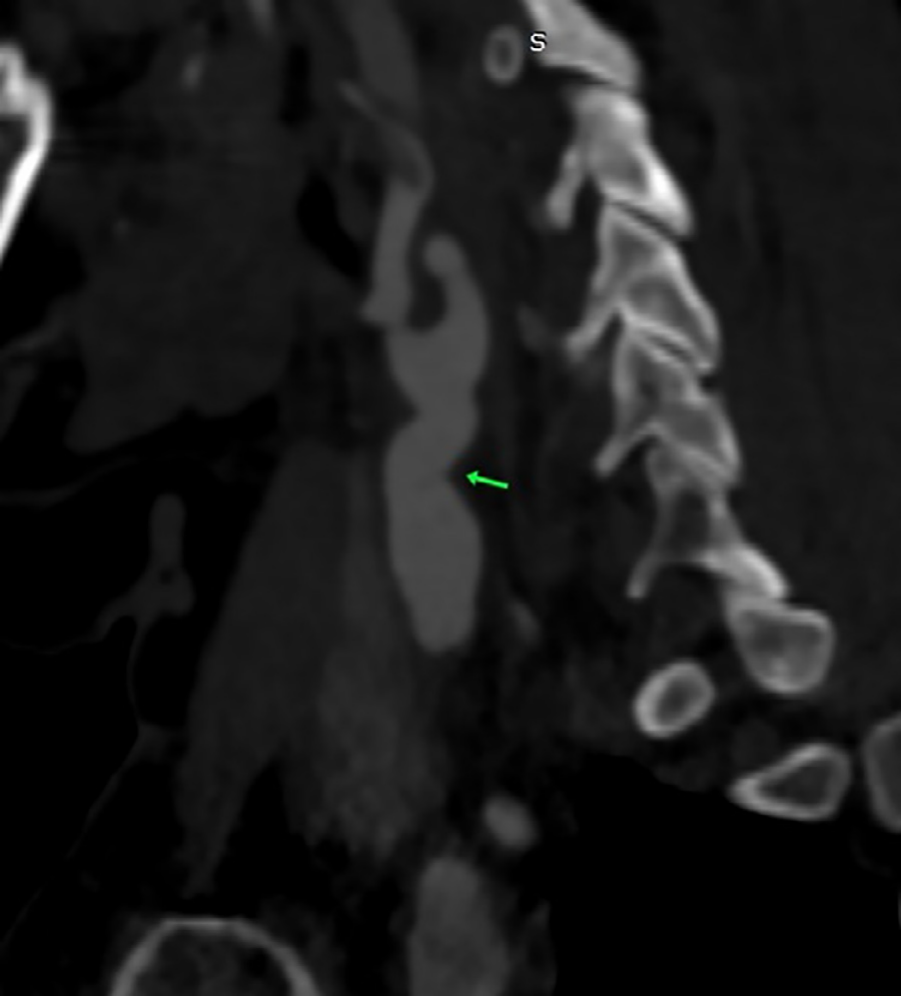
CTA showed a transverse, low density strip (arrow), causing luman stenosis, with proximal part aneurysmal enlargement on lower part of right CCA.

A carotid stent placement was refused by the patient. The aneurysm location in the proximal CCA increased the technical difficulty of a carotid endarterectomy operation. Medical therapy with anti-platelet (aspirin or clopidogrel bisulfate) was a good alternative.

Our patient benefits from a regular follow up and experienced no more neurologic effects.

## Discussion

After searching in the MEDLINE/Pub Med/Embase databases using combinations of the keywords diaphragm, common carotid, carotid stenosis, and atypical fibro muscular dysplasia (FMD), there were only two case reports of CCA diaphragm reported discovered by ultrasound reported in the literature [1,2].

Carotid diaphragms are a non-atheromatous arterial cause of ischemic stroke, especially in people under 65 years old. This anomaly is more common in the African or African-American population [3]. This cause of cerebro-vascular accident (CVA) results from a non-atheromatous overgrowth of the intima of the arterial wall which appears in imagery as an endoluminal web causing carotid stenosis [3,4]. Patients with carotid diaphragms can lead to locally formed thrombi that can cause recurrent embolic stroke [4].

Carotid diaphragm has been considered as a subtype of FMD with non atherosclerotic and non-inflammatory histopathology. FMD is a disease of the musculature of the arterial wall that leads to aneurysm, dissection, stenosis or occlusion [5], [6], [7].

Intimal dissection and atheromatous plaque are the two main differential diagnoses of the carotid diaphragm. However, the appearance of a membrane implanted on a regular non atheromatous wall and the absence of any other localization of atheroma distinguish au authentic diaphragm from a focal atheromatous plate. In addition, the very proximal localization of the diaphragm, in the CCA, does not suggest a dissection, which is usually localized downstream of the bulb [6].

Carotid diaphragm mainly occurs in the ICA and the vertebral artery^.^. All carotid diaphragms were typically in the carotid bulb or in the proximal-to-middle segment of the ICA. Only two case reports of CCA diaphragms have been reported [1,8].

Most carotid diaphragms reported earlier were initially diagnosed by CDU. The characteristic of ultrasound finding is described as a membranous appearance or a linear band tissue. We report a case of carotid diaphragm, atypical by its rare localization at the level of the CCA on one hand and by the association with an aneurysm of the downstream carotid on the other hand. In addition, aneurysm is not common in the extra-cranial carotid artery caused by the carotid diaphragm [8]. In fact, we believe that the local CCA stenosis may cause localized resistance of blood vessels, hemodynamic disturbance and high pressure on the vessel wall which can lead to its widening.

Management of carotid diaphragm varies depending on patient's symptoms. Medical treatment with antiplatelet is typically used for asymptomatic patients. For several symptomatic patients, Carotid endarterectomy or carotid artery stenting can been proposed as an alternative treatment. The anatomic aneurysm location in the proximal to the middle of CCA in our patient made this alternative technically difficult.

### Conclusion

We presented an unusual case of CCA's diaphragm with an accompanying aneurysm, first detected by CDU and subsequently confirmed by CTA.

To our knowledge, this is the third reported CCA diaphragm that is also possible FMD.

This case detailed ultrasound features of carotid diaphragm, so that clinicians can be more mindful with such ultrasound findings in the future particularly while exploring syncope in young adults.

## References

[1] Wang Y, Li JC, Lv K, Wang NL, Sharen GW, Tan L. (2017). A rare diaphragm in the common carotid artery: a first case report and literature review. Medicine (Baltimore).

[2] Liu Y, Hua Y, Ling C, Lei N, Feng W. (2017). Atypical common carotid artery diaphragm with an accompanying aneurysm. Am J Med Sci.

[3] Hennequin JL, Molinié V, Smadja D, Touzé E, Olindo S (2014). Carotid-bulb atypical fibromuscular dysplasia in young Afro-Caribbean patients with stroke. Stroke.

[4] Lenck S., Labeyrie M.-A., Saint-Maurice J.-P., Tarlov N., Houdart E. (2014). Diaphragms of the carotid and vertebral arteries: an under-diagnosed cause of ischaemic stroke. Eur J Neurol.

[5] Touzé E, Oppenheim C, Trystram D (2010). Fibromuscular dysplasia of cervical and intracranial arteries. International Journal of Stroke.

[6] Haussen DC, Grossberg JA, Bouslama M, Pradilla G, Belagaje S, Bianchi N (2017). Carotid Web (Intimal Fibromuscular Dysplasia) has high stroke recurrence risk and is amenable to stenting. Stroke.

[7] Lenck S, Labeyrie MA, Saint-Maurice JP, Tarlov N, Houdart E. (Apr 2014). Diaphragms of the carotid and vertebral arteries: an under-diagnosed cause of ischaemic stroke. Eur J Neurol.

[8] Liu Y, Hua Y, Ling C, Lei N, Feng W. (2017). Atypical common carotid artery diaphragm with an accompanying aneurysm. Am J Med Sci.

